# What cellular mechanisms are related to thromboembolic events in patients with COVID-19?

**DOI:** 10.1590/1677-5449.210020

**Published:** 2021-08-02

**Authors:** Cristian Rodrigues do Nascimento, Júlio Martinez Santos, Sávio Breno Pires Brito, Pedro Pereira Tenório

**Affiliations:** 1 Universidade Federal do Vale do São Francisco – UNIVASF, Paulo Afonso, BA, Brasil.; 2 Universidade Federal de São Paulo – UNIFESP, São Paulo, SP, Brasil.

**Keywords:** coronavirus infections, disseminated intravascular coagulation, blood coagulation

## Abstract

SARS-CoV-2 is the virus responsible for the COVID-19 pandemic. This disease is beginning to be better understood in terms of its other, non-respiratory, clinical manifestations. Over the course of months caring for patients infected by the virus, clinical and laboratory changes have been identified that have prompted researchers to debate the potential that SARS-CoV-2 has to trigger an exacerbated immune response that is capable of changing endothelial homeostasis through both direct and indirect mechanisms. With the intention of contributing to this debate, a review was conducted of the possible mechanisms that could trigger these phenomena in patients with COVID-19. It is important to understand the pathophysiology of the immunological mechanisms related to this disease in order to understand the potential endothelial damage that COVID-19 can provoke.

## INTRODUCTION

Novel coronavirus disease (COVID-19) has been linked to coagulopathies, both via direct mechanisms and via indirect mechanisms, secondary to development of exacerbated inflammation, known as a cytokine storm.^1^ This disease is caused by the severe acute respiratory syndrome coronavirus 2 (SARS-CoV-2), which is a member of the Betacoronavirus genus.^2^ SARS-CoV-2 contain several different structural and non structural proteins that are essential for its survival and perpetuation.^2^ Its structural proteins have a number of different functions, and the most important of them is the spike (S) protein, which is itself divided into two subunits.^2^

Subunit S1 is responsible for binding to angiotensin-converting enzyme II (ACE-II) and subunit S2 modulates the mechanism of viral fusion with the host cell membrane.^2^ The viral envelope is made up of the membrane protein (M) and the envelope protein (E), which, in conjunction, give the virus greater protection against external agents.^2^ The hemagglutinin esterase (HE) protein aids the bonding/invasion mechanism and the nucleocapsid protein (N) is involved in regulation of replication.^2^^,^^3^ Mature non-structural proteins (NSPs) take part in a variety of functions that are important to the process of viral dissemination, such as, for example, the viral replication process.^3^

## METHODOLOGY

This is a descriptive review of the literature based on searches using the Portuguese keywords: “*infecções por coronavírus*” AND “*coagulação intravascular disseminada*” AND/OR “*coagulação sanguínea*” and the English terms: “*coronavirus infections*” AND “*disseminated intravascular coagulation*” AND/OR “*blood coagulation*”. Since this is a recent disease, the searches were run without date limits, between October 2020 and February 2021. A large number of studies were analyzed, but only twelve of these were chosen as meeting the inclusion criteria, which were to present some type of molecular or biochemical mechanism of endothelial injury caused by infection by SARS-CoV-2 and/or discuss the thromboembolic process in patients with COVID-19. Since this is a descriptive bibliographic review, the aim was to compile the maximum quantity of scientific evidence (Table 1) that could be presented in a brief communication on the possible cellular mechanisms involved in thromboembolic events in patients with COVID-19.

**Table 1:** Evidence levels of the bibliographic references, assessed according to the Oxford Center for Evidence-based Medicine classification system.

**Reference number**	**Article title**	**Area of study**	**Evidence level**
1	COVID19 and thrombotic or thromboembolic disease: implications for prevention, antithrombotic therapy, and follow-up	Prognosis	5
2	A comparison of COVID-19, SARS and MERS	Diagnosis	3B
3	Epidemiology, virology, and clinical features of severe acute respiratory syndrome-coronavirus-2 (SARS-CoV-2; Coronavirus Disease-19)	Treatment/prevention/etiology/injury	5
4	SARS-CoV-2 binds platelet ACE2 to enhance thrombosis in COVID-19	Treatment/prevention/etiology/injury	2B
5	Coagulation dysfunction in COVID-19: the interplay between inflammation, viral infection and the coagulation system	Diagnosis	3B
6	O coração e a COVID-19: o que o cardiologista precisa saber [The heart and COVID-19: what the cardiologist needs to know]	Prognosis	**5**
7	Endothelial cell infection and endotheliitis in COVID-19	Treatment/prevention/etiology/injury	4
8	COVID-19, immune system response, hyperinflammation and repurposing antirheumatic drugs	Treatment/prevention/etiology/injury	2C
9	Severe Acute Respiratory Syndrome Coronavirus 2 (SARS-CoV-2): An overview of viral structure and host response	Treatment/prevention/etiology/injury	5
10	Fisiopatologia da trombose associada à infecção pelo SARS-CoV-2 [Pathophysiology of thrombosis associated with SARS-CoV-2 infection]	Treatment/prevention/etiology/injury	5
11	COVID-19 and its implications for thrombosis and anticoagulation	Treatment/prevention/etiology/injury	4
12	Coagulopathy of Coronavirus Disease 2019	Treatment/prevention/etiology/injury	4

### Direct vascular endothelial injury

SARS-CoV-2 can directly cause a process of endotheliitis in many organs, which is an event that has been demonstrated by identification of viral bodies inside these cells and consequent inflammatory response, resulting in cell death.^3^ Endotheliitis explains the vascular manifestations seen in severe cases of COVID-19, providing a foundation for possible new treatment approaches, such as, for example, use of drugs to stabilize the endothelium while the immune system acts in the process of eliminating the virus from the organism.^3^

**Figure 1:**
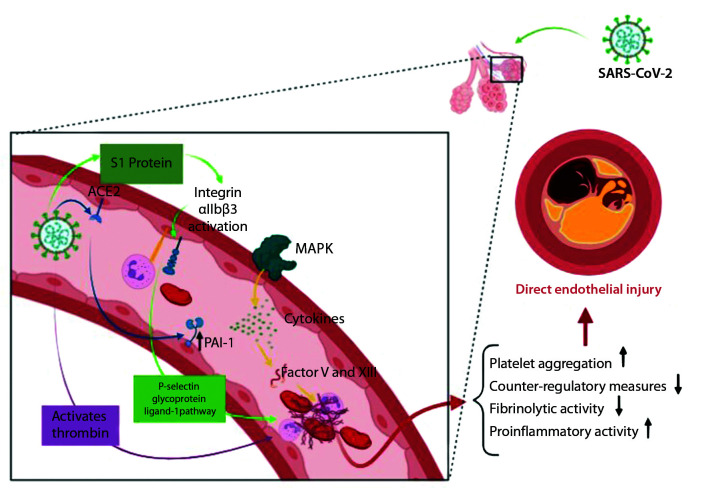
Direct endothelial injury pathways mediated by the severe acute respiratory syndrome coronavirus-2 (SARS-CoV-2). ACE2 = angiotensin-converting enzyme II; Protein S1 = Spike protein 1; MAPK = mitogen activated protein kinase; PAI-1 = plasminogen activator inhibitor type 1; Factor V = proaccelerin; Factor XIII = fibrin stabilizing factor.

Several different mechanisms to explain the processes involved in direct injury have been postulated (Figure 1). Those that have been studied most include the following:

I – The S protein increases platelet aggregation and adenosine triphosphate consumption in a directly proportional manner. More specifically, the S1 subunit, but not S2, is capable of inducing the platelet-mediated coagulation process. Analyses conducted with flow cytometry led to the discovery that the S1 subunit also induces increased integrin αIIbβ3 activation and increases P-selectin expression.^4^ Integrin αIIbβ3 is the major structural protein in platelets and is responsible for platelet aggregation when it transitions from its resting state to its active state, binding to other integrins. It is known that this protein performs an important function in interactions between platelets and leukocytes, via the P-selectin glycoprotein ligand-1pathway, causing large-scale production of platelet-leukocyte aggregates, which play an important role in endothelial injury and, as a consequence, in formation of thrombi.^4^
II – The direct viral aggression mechanism is also related to activation of mitogen activated protein kinase (MAPK), which is responsible for production and modulation of the cytokines induced by SARS-CoV-2 in pulmonary cells.^4^ MAPK potentiates platelet aggregation and reduces the counter-regulatory measures responsible for retraction of the clots thus produced. SARS-CoV-2 induces thrombin activation directly and induces platelets to release coagulation factors V and XIII.^4^
III – Another mechanism is binding of the virus itself to ACE-II. This causes a substantial reduction in the quantity of this protein free to perform its functions, with a resultant increase in availability of angiotensin II, increasing production of plasminogen activator inhibitor I (PAI1), reducing fibrinolytic activity and causing an imbalance between coagulation regulation mechanisms.^5^^,^ 6
IV – Finally, SARS-CoV-2 is capable of infecting endothelial cells, replicating them in an uncontrolled manner, and causing cell death, resulting in hyperactivation of procoagulatory reactions in severe COVID-19 cases.^6^ Attraction of immune cells to the site of invasion/lesion, both directly and by chemotactic mechanisms, causes further disseminated endothelial injury which may result in changes to microvascular homeostasis, in the direction of vasoconstriction and subsequent ischemia of many different structures, in addition to inflammation and hypercoagulability.^7^

### Indirect vascular endothelial injury

Viral infection of the respiratory epithelium has the potential to trigger an inflammatory process, which may be controlled or uncontrolled. When uncontrolled, it can cause hypercoagulability.^8^ This state can lead to thrombotic events, which are intensified by exacerbated production of proinflammatory cytokines such as interferon α and γ, interleukins 1β, 6, 12, 18, and 33, tumor necrosis factors α and β, granulocyte colony-stimulating factor and macrophages.^8^^,^^9^

**Figure 2:**
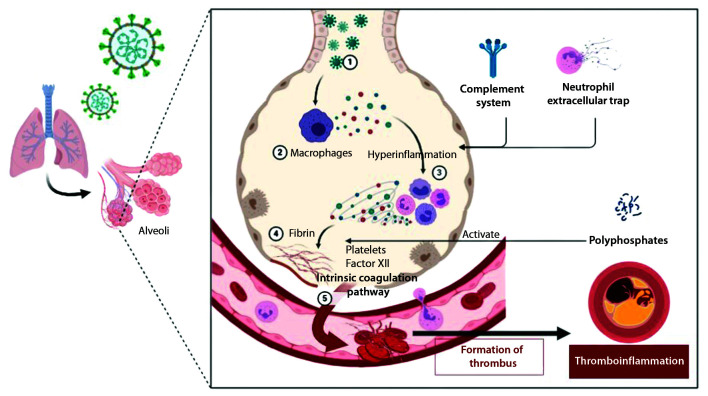
Mechanism of indirect endothelial injury caused by the severe acute respiratory syndrome coronavirus 2 (SARS-CoV-2), mediated by a proinflammatory cytokine storm. 1 = SARS-COV-2; 2 = Macrophages; 3 = Proinflammatory cytokines leading to a process of vascular hyperinflammation; 4 = Formation of fibrin networks; 5 = Activation of the intrinsic coagulation pathway, triggering thrombi formation and displacing this activity to thromboinflammation involving many different blood vessels.

The complex inflammatory responses trigger a procoagulatory response via several pathways, with consequent thrombin production. 10 Polyphosphates that are released by the microorganism activate mast cells, platelets, and coagulation factor XII and also activate other pathways related to the intrinsic coagulation pathway.^10^^,^^11^ The complement system and neutrophil extracellular traps also stimulate thrombin production.^10^^,^^11^ This procoagulatory response constitutes an essential component of communication of humoral and cellular responses, amplifying the immune response, in a process known as thromboinflammation (Figure 2).^11^ This leads to increased risk of thrombotic complications, which is more prominent in individuals who enter a severe inflammatory state.^12^ Such patients are more predisposed to deteriorating clinical status, including pulmonary embolism and microvascular thrombosis of the lungs.^10^^,^^12^

## CONCLUSIONS

The inflammatory response triggered by SARS-CoV-2 infection can lead a series of endothelial events that have repercussions for hemostasis. It is therefore evident that further studies are needed to acquire more exact knowledge about the molecular mechanisms involved in the thromboembolic events that occur in patients with severe COVID-19. Recognition of those who have a greater predisposition to procoagulatory states enables prognoses to be established and better management approaches to be developed, particularly for more severe patients, such as those with systemic infections and high morbidity and mortality rates, and those with preexisting endothelial injury.
