# Supplementary material for: What cellular mechanisms are related to thromboembolic events in patients with COVID-19?
Source: J Vasc Bras. 2021 Aug 2;20:e20210020. doi: 10.1590/1677-5449.210020 (PMC8340722; doi:10.1590/1677-5449.210020)
Supplement: Translation

# Translation: Quais são os mecanismos celulares relacionados aos eventos tromboembólicos em pacientes com COVID-19?

**Authors:** Cristian Rodrigues do Nascimento, Júlio Martinez Santos, Sávio Breno Pires Brito, Pedro Pereira Tenório

**Affiliations:** 1 Universidade Federal do Vale do São Francisco – UNIVASF, Paulo Afonso, BA, Brasil.; 2 Universidade Federal de São Paulo – UNIFESP, São Paulo, SP, Brasil.

**Keywords:** infecções por coronavírus, coagulação intravascular disseminada, coagulação sanguínea

## Abstract

O SARS-CoV-2 é o vírus responsável pela pandemia da COVID-19. Essa doença começou a ser melhor entendida devido a outras manifestações clínicas além das respiratórias. Ao longo dos meses de atendimento aos pacientes infectados pelo vírus, foram identificada**s** alterações clínicas e laboratoriais que incitaram os pesquisadores a discutir sobre o potencial do SARS-CoV-2 no desencadeamento de uma resposta imunológica exacerbada capaz de alterar a homeostase endotelial, através de mecanismos diretos e indiretos. Com esse intuito, foram revisados os possíveis mecanismos que desencadeiam este fenômeno em pacientes portadores de COVID-19. Dessa forma, torna-se importante o entendimento fisiopatológico dos mecanismos imunológicos relacionados à doença para a compreensão do potencial de dano endotelial que a COVID-19 pode promover.

## INTRODUÇÃO

A doença pelo novo coronavírus (COVID-19) tem sido associada a coagulopatias, tanto por mecanismos diretos quanto indiretos, secundárias ao desenvolvimento de uma inflamação exacerbada, chamada de tempestade de citocinas^1^. Essa doença é causada pelo coronavírus 2 da síndrome respiratória aguda grave (SARS-CoV-2), pertencente ao gênero Betacoronavírus^2^. O SARS-CoV-2 apresenta diferentes proteínas essenciais estruturais e não estruturais para a sua sobrevivência e perpetuação^2^. As proteínas estruturais apresentam diferentes funções, destacando-se a proteína *spike* (S), que é dividida em duas subunidades^2^.

A subunidade S1 é responsável pela ligação à enzima conversora de angiotensina II (ECA-II), e a subunidade S2 modula o mecanismo de fusão viral com a membrana celular do hospedeiro^2^. O envelope viral é constituído pela proteína de membrana (M) e a proteína de envelope (E), que, em conjunto, garantem maior proteção do vírus contra agentes externos^2^. A proteína hemaglutinina esterase (HE) auxilia no mecanismo de ligação/invasão, e a proteína do nucleocapsídeo (N) está envolvida na regulação da replicação^2^^,^^3^. As proteínas não estruturais maduras (NSPs) participam de diversas funções importantes para o processo de disseminação viral, como, por exemplo, o processo de replicação do vírus^3^.

## METODOLOGIA

Trata-se de uma revisão da literatura do tipo descritiva, que utilizou como palavras-chave os descritores em português: “*infecções por coronavírus*” AND “*coagulação intravascular disseminada*” AND/OR “*coagulação sanguínea*”, assim como foram usados os termos em língua inglesa: “*Coronavirus Infections*” AND “*Disseminated Intravascular Coagulation*” AND/OR “*Blood Coagulation*”. Por ser uma doença recente, a busca se deu sem restrição de tempo, sendo realizada entre os meses de outubro de 2020 a fevereiro de 2021. Foram analisados diversos estudos; entretanto, desses, apenas 12 foram escolhidos, pois atendiam aos critérios de inclusão, os quais apresentavam algum mecanismo molecular ou bioquímico de lesão endotelial decorrente da infecção do SARS-CoV-2 e/ou discutiam sobre o processo tromboembólico em pacientes com COVID-19. Por se tratar de uma revisão bibliográfica descritiva, o intuito deste trabalho foi reunir o máximo de evidências científicas (Tabela 1) que pudessem ser apresentadas em uma breve comunicação sobre os possíveis mecanismos celulares envolvidos nos eventos tromboembólicos de pacientes com COVID-19.

**Tabela 1:** Níveis de evidência das referências bibliográficas avaliados de acordo com o sistema de classificação do Centro de Medicina Baseada em Evidências de Oxford.

**Número da referência**	**Título do artigo**	**Área do estudo**	**Nível de evidência**
1	COVID19 and thrombotic or thromboembolic disease: implications for prevention, antithrombotic therapy, and follow-up	Prognóstico	5
2	A comparison of COVID-19, SARS and MERS	Diagnóstico	3B
3	Epidemiology, virology, and clinical features of severe acute respiratory syndrome-coronavirus-2 (SARS-CoV-2; Coronavirus Disease-19)	Terapia/prevenção/etiologia /danos	5
4	SARS-CoV-2 binds platelet ACE2 to enhance thrombosis in COVID-19	Terapia/prevenção/etiologia/danos	2B
5	Coagulation dysfunction in COVID-19: the interplay between inflammation, viral infection and the coagulation system	Diagnóstico	3B
6	O coração e a COVID-19: o que o cardiologista precisa saber	Prognóstico	**5**
7	Endothelial cell infection and endotheliitis in COVID-19	Terapia/prevenção/etiologia/danos	4
8	COVID-19, immune system response, hyperinflammation and repurposing antirheumatic drugs	Terapia/prevenção/etiologia/danos	2C
9	Severe Acute Respiratory Syndrome Coronavirus 2 (SARS-CoV-2): An overview of viral structure and host response	Terapia/prevenção/etiologia/danos	5
10	Fisiopatologia da trombose associada à infecção pelo SARS-CoV-2	Terapia/prevenção/etiologia/danos	5
11	COVID-19 and its implications for thrombosis and anticoagulation	Terapia/prevenção/etiologia/danos	4
12	Coagulopathy of Coronavirus Disease 2019	Terapia/prevenção/etiologia/danos	4

### Lesão direta ao endotélio vascular

O SARS-CoV-2 pode levar diretamente a um processo de endotelite em vários órgãos, evento já estabelecido pela identificação de corpos virais dentro dessas células e da consequente resposta inflamatória resultante da morte celular^3^. A endotelite explica as manifestações vasculares existentes nos casos graves da COVID-19, o que subsidia a possibilidade de novas abordagens terapêuticas, como, por exemplo, o uso de drogas estabilizantes do endotélio enquanto o sistema imune atua no processo de eliminação do vírus no organismo^3^.

Foram postulados diferentes mecanismos (Figura 1) que explicam os processos de lesão direta. Entre esses, os mais estudados são:

**Figura 1:** Vias de lesão endotelial direta mediada pelo coronavírus 2 da síndrome respiratória aguda grave (SARS-CoV-2). ECAII = enzima conversora de angiotensina II; proteína S1 = proteína *Spike* 1; MAPK = proteína quinase ativada por mitógenos; PAI-1 = inibidor do ativador do plasminogênio tipo 1; fator V = proacelerina; fator XIII = fator estabilizador da fibrina.

I - A proteína S aumenta de forma diretamente proporcional a agregação plaquetária e o consumo de adenosina trifosfato. De forma mais específica, a subunidade S1, mas não a S2, apresenta a capacidade de induzir o processo de coagulação mediada por plaquetas. A partir de análises em citometria de fluxo, foi descoberto também que a subunidade S1 induz a maior ativação da integrina αIIbβ3 e a maior expressão da P-selectina^4^. A integrina αIIbβ3 é a principal proteína estrutural das plaquetas e a responsável pela agregação plaquetária ao passar do seu estado de repouso para o seu estado ativo, realizando, assim, ligação com outras integrinas. Sabe-se que essa proteína tem importante função na interação entre plaquetas e leucócitos pela via P-selectina glicoproteína ligante 1, resultando, dessa forma, em uma grande produção de agregados de leucócitos-plaquetas que apresentam forte papel na lesão endotelial e, por consequência, na formação de trombos^4^.
II - O mecanismo de agressão direta do vírus também está relacionado à ativação da proteína quinase ativada por mitógenos (MAPK), que é responsável pela produção e modulação de citocinas induzidas pelo SARS-CoV-2 em células pulmonares^4^. A MAPK tem efeito potencializador na agregação plaquetária e diminui os mecanismos contrarreguladores responsáveis pela retração dos coágulos produzidos. O SARS-CoV-2 induz diretamente a ativação da trombina e as plaquetas a liberarem o fator V e o XIII da coagulação^4^.
III - Outro mecanismo é a própria ligação do vírus a ECA-II. Através disso, a quantidade dessa proteína livre para exercer suas funções é reduzida de maneira substancial, resultando em uma maior disponibilidade de angiotensina II, o que favorece o aumento da produção de inibidor do ativador de plasminogênio I (PAI1), levando a diminuição da atividade fibrinolítica e a um desbalanço nos mecanismos de regulação da coagulação^5^^,^ 6.
IV – Por fim, o SARS-CoV-2 é capaz de infectar células endoteliais, replicá-las de maneira descontrolada e causar a morte celular, resultando em hiperativação de reações pró-coagulantes em casos graves da COVID-19^6^. A atração das células imunes para o local de invasão/lesão, tanto por via direta quanto por mecanismo**s** quimiotáticos, resulta em mais lesões endoteliais disseminadas, que podem determinar uma alteração na homeostase microvascular em direção à vasoconstrição e subsequente isquemia de diversas estruturas, além da inflamação e hipercoagulabilidade^7^.

### Lesão indireta ao endotélio vascular

Ao infectar o epitélio respiratório, o vírus tem potencial para gerar um processo inflamatório, o qual pode ocorrer de forma controlada ou não. Quando descontrolada, pode resultar em hipercoagulabilidade^8^. Tal estado pode conduzir a eventos trombóticos, que são intensificados pela produção exacerbada de citocinas pró-inflamatórias como interferon α e γ, interleucinas 1β, 6, 12, 18 e 33, fator de necrose tumoral α e β, fator estimulador de colônias de granulócitos e macrófagos^8^^,^ 9.

As complexas respostas inflamatórias levam a uma resposta pró-coagulante, oriunda de muitas vias, com consequente geração de trombina^10^. Os polifosfatos que são liberados pelo microrganismo levam à ativação de mastócitos, plaquetas e fator XII da coagulação, além de ativarem outras vias relacionadas à via intrínseca da coagulação^10^^,^ 11. O sistema complemento e as armadilhas extracelulares dos neutrófilos também levam à geração de trombina^10^^,^ 11. Essa resposta pró-coagulante atua como componente essencial na comunicação das respostas humoral e celular na amplificação da resposta imune, sendo este processo denominado de tromboinflamação (Figura 2)^11^. Isso leva a um aumento do risco de complicações trombóticas, com mais destaque para aqueles indivíduos que apresentam quadro inflamatório grave^12^. Esses pacientes estão mais predispostos a sofrerem agravos do quadro clínico, como embolia pulmonar e trombose microvascular nos pulmões^10^^,^ 12.

**Figura 2:** Mecanismo de lesão endotelial indireta ocasionado pelo coronavírus 2 da síndrome respiratória aguda grave (SARS-CoV-2), mediado pela tempestade de citocinas pró-inflamatórias. 1 = SARS-COV-2; 2 = Macrófagos; 3 = Citocinas pró-inflamatórias levando ao processo de hiperinflamação vascular; 4 = Formação das redes de fibrina; 5 = Ativação da via intrínseca da coagulação que desencadeia a formação de trombos deslocando essa ação para uma tromboinflamação que acomete diversos vasos sanguíneos.

## CONCLUSÃO

A resposta inflamatória desencadeada pela infecção por SARS-CoV-2 pode levar a uma série de eventos endoteliais que repercutem na hemostasia. Dessa forma, fica evidente que são necessários mais estudos que permitam conhecer com maior exatidão os mecanismos moleculares envolvidos com os eventos tromboembólicos nos pacientes graves com COVID-19. Associado ao reconhecimento daqueles com maior predisposição para o estado pró-coagulante, é possível estabelecer prognósticos e desenvolver melhores condutas, principalmente frente aos pacientes mais graves, como portadores de infecções sistêmicas e com alto grau de morbimortalidade, assim como naqueles com lesão endotelial prévia.
